# Immunoglobulin M response in largemouth bass (*Micropterus salmoides*) following ranavirus infection

**DOI:** 10.3389/fimmu.2025.1515684

**Published:** 2025-01-29

**Authors:** Zhenyu Huang, Naicheng Liu, Mingyang Xue, Chen Xu, Yuding Fan, Yan Meng, Nan Jiang, Yiqun Li, Wenzhi Liu, Yang He, Yong Zhou

**Affiliations:** ^1^ Yangtze River Fisheries Research Institute, Chinese Academy of Fishery Sciences, Wuhan, Hubei, China; ^2^ Key Laboratory of Sichuan Province for Fishes Conservation and Utilization in the Upper Reaches of the Yangtze River, Neijiang Normal University, Neijiang, Sichuan, China

**Keywords:** IgM^+^ B cells, immersion infection, pathological changes, virus-specific IgM, immune response

## Abstract

Immunoglobulin M (IgM) and IgM^+^ B cells are key components of the humoral immune system, providing defense against pathogen invasion. While the role of IgM in the systemic and mucosal immune responses of fish to parasites and bacteria has been partially investigated, its function in viral infections remains underexplored. This study successfully developed a largemouth bass (*Micropterus salmoides*) model for ranavirus immersion infection. Our findings revealed that viral infection caused significant pathological changes in the gill and head kidney tissues, along with a marked upregulation of adaptive immune gene expression. Interestingly, fish that survived an initial viral infection exhibited minimal mortality and low viral loads in the gill and head kidney tissues when exposed to a higher viral concentration. Notably, in these fish with secondary infections, there was a significant increase in IgM protein levels in both the blood and gill mucus, as well as a pronounced accumulation of IgM^+^ B cells in the gill and head kidney tissues. Additionally, the serum contained high levels of virus-specific IgM, which demonstrated the ability to neutralize the virus. These findings highlight the crucial role of IgM in the immune response to viral infections in largemouth bass and suggest its potential as a target for enhancing viral resistance in aquaculture.

## 1 Introduction

Teleost fish inhabit diverse aquatic ecosystems rich in microbial communities (1). To thrive in these environments, they have evolved a sophisticated immune system comprising both innate and adaptive components (2). In mammals, B cells play a pivotal role in coordinating innate and adaptive immune responses (3). Like macrophages and neutrophils, B cells can perform phagocytosis, using B cell receptors to recognize distinct antigen epitopes and presenting them to CD4^+^ T cells to trigger an adaptive immune response (4). Upon antigen stimulation and CD4^+^ T cell activation, B cells can proliferate and differentiate into plasma cells (5), which then secrete specific antibodies to neutralize or eliminate pathogens (6). Similar to mammals, fish exhibit a diversity of B cell populations, including plasma cells, plasmablasts, and memory B cells. Plasmablasts are early-stage cells that differentiate from B cells and are primarily produced during the initial phase of an immune response. Plasma cells represent a mature immune cell type that arises from the further differentiation of plasma blasts. Memory B cells are B cells that survive the initial immune response and can persist for an extended period. While there are no specific antibodies that can distinguish these B cells, several B cell-specific transcription factors, including paired box-5 and B lymphocyte-induced protein-1, have been identified for the purpose of studying teleost B cell development (7, 8). Teleost B cells exhibit similar functions to mammalian B cells, including phagocytosis, antigen presentation, and the secretion of immunoglobulins (Igs) aimed at resisting pathogen invasion (9–11).

The principal components of the humoral adaptive immune response are the Igs (12). Igs are a class of glycoproteins with remarkable abilities to recognize a wide range of antigens, including those from bacteria, viruses, and other pathogens (13). They can also recruit other cells and molecules to aid in pathogen neutralization (14). In teleost fish, three known isotypes of the Ig heavy chain have been identified: IgM, IgD, and IgT/Z (15). IgM was the first Ig discovered in fish and is the most abundant immunoglobulin in plasma (16). It can exist as a secreted antibody or as a transmembrane surface protein (10). Similar to higher vertebrates, teleost IgM performs various effector functions in humoral immune responses, such as activating the complement system, promoting agglutination, binding to mannose-binding lectin motifs, and inducing cellular cytotoxicity (17–20). Additionally, studies have shown that teleost IgM can enhance the phagocytic activity of macrophages through antigen-specific opsonization (21). Secreted IgD has been observed to cover a significant portion of the fish microbiome (22, 23), though its role in immune defense remains largely unexplored. IgT, a unique immunoglobulin of bony fish, was first identified in 2005 in rainbow trout (*Oncorhynchus mykiss*) and zebrafish (*Danio rerio*) (24, 25). IgT has been shown to function as a mucosal-specific immunoglobulin (10), with increased IgT synthesis observed at mucosal surfaces such as the gut, gills, and skin in response to parasitic infections. In contrast, changes in IgM levels are restricted to systemic compartments (10, 26, 27). The three Ig isotypes have been shown to define four distinct B cell subsets: IgM^+^, IgD^+^, IgT^+^, and IgM^+^IgD^+^. It is noteworthy that infection with parasites or bacteria resulted in a pronounced elevation in the number of IgM^+^ B cells within systemic immune tissues, whereas only minimal alterations were discerned in mucosal tissues (28). In contrast, viral infections or viral vaccine-induced responses resulted in disparate alterations in the number of IgM^+^ B cells in mucosal tissues. For example, when the virus infected rainbow trout, there was no statistically significant change in the number of IgM^+^ B cells in the swim bladder mucosa (15). Nevertheless, in carp (*Cyprinus carpio*) infected with the virus, a notable increase in IgM^+^ B cells was observed in the intestinal mucosa (29). While research on B cells and Igs in teleost fish has been more extensive in certain species such as rainbow trout and zebrafish, studies on other fish species remain relatively limited.

The largemouth bass is an important species in Chinese aquaculture. Following the development of artificial compound feed, the scale and yield of largemouth bass farming in China have grown rapidly (30). However, the intensification of aquaculture has increased the risk of disease outbreaks, particularly viral infections caused by the largemouth bass ranavirus (LMBRaV) (31). LMBRaV infection in largemouth bass leads to patchy hemorrhagic ulcers on the body surface, along with redness, swelling, and ulceration of the caudal peduncle. The liver often becomes enlarged and whitish or yellowish in appearance, and in some cases, the spleen is also enlarged, though the body surface remains intact (32, 33). Several studies have been conducted to evaluate the effectiveness of vaccines in protecting against LMBRaV (34–36). A thorough understanding of the humoral adaptive immune response is crucial for developing effective vaccines (37). However, research into the humoral adaptive immune response in largemouth bass remains limited.

The objective of this study was to assess the adaptive immune response, particularly focusing on IgM^+^ B cells and the IgM response, in the context of LMBRaV infection. Largemouth bass were exposed to the virus via bath immersion. Our findings showed that the gills and head kidney (HK) exhibited strong adaptive immune responses to LMBRaV, with the IgM^+^ B cells and IgM response being more prominent in the HK than in the gills. Additionally, IgM was shown to neutralize the virus, as demonstrated by IgM-depletion experiments. In conclusion, these results highlight the vital role of the HK and gills in mounting a robust humoral response against viral infections in largemouth bass. The ability of IgM to neutralize the virus underscores its importance in the adaptive immune response and suggests potential strategies for improving disease resistance in largemouth bass through selective breeding or immunization.

## 2 Materials and methods

### 2.1 Fish maintenance

The experimental population of largemouth bass, with an average body weight of 6.5 ± 1.0 g, was sourced from Huangpi (Hubei, China) and housed in a recirculating aquaculture system with aeration and an internal biofilter at a temperature of 28°C. The fish received commercial compound feed bi-daily and experienced a two-day fasting interval before infection. All animal procedures were approved by the Animal Experiment Committee of the Yangtze River Fisheries Research Institute and conducted in compliance with applicable guidelines.

### 2.2 Viral infection

LMBRaV was previously isolated from sick largemouth bass in Hubei Province (38) and propagated in epithelioma papulosum cyprinid (EPC) cells cultured in minimum essential medium (MEM) enriched with 2% fetal bovine serum (FBS) at 28°C. Following the appearance of extensive cytopathic effects (CPE), the EPC cells infected with LMBRaV were harvested and subjected to three freeze-thaw cycles to produce a viral suspension. The resultant supernatant was then serially diluted (10^−1^ to 10^−12^) and inoculated into 96-well cell culture plates containing EPC cells at 90% confluence. To determine the tissue culture infectious dose (TCID50) of LMBRaV, the CPE were observed at 24-hour intervals over a 7-day period. The virus stock was subsequently stored at −80°C until required for use.

Two distinct exposure protocols to LMBRaV were employed for the infection experiment, resulting in two groups of fish. The first group was exposed to the virus once, while the second group was exposed twice. The first group of largemouth bass was immersed in a solution containing 40 mL of LMBRaV (3.5 × 10^7^ TCID50) in 20 L of aerated water for 4 hours at 28°C. Following this period, the fish were transferred to aquaria containing fresh water. The control fish were treated identically but with MEM instead of the virus. In the second exposure protocol, fish that had survived the primary infection were re-challenged with the double doses of LMBRaV at 21 dpi.

### 2.3 Sample collection

At 1, 4, 7, 14, and 21 days post-infection (dpi), the fish were euthanized using an overdose of tricaine methanesulfonate (MS-222, Sigma), and tissues, including HK and gill, were collected for analysis of viral loads, pathological changes, and immune gene expression. At 28 dpi, samples of HK and gill tissues were taken in order to quantify B cell numbers, and serum and gill mucus were collected for the purpose of analyzing changes in IgM protein levels, LMBRaV-specific IgM titers, and neutralizing virus experiment.

### 2.4 Real-time quantitative PCR analysis

Total RNA was isolated from HK and gill tissues with TRIzol Reagent (Invitrogen) in accordance with the manufacturer’s protocol. The RNA concentration was quantified using spectrophotometry (Nanodrop ND1000), and RNA integrity was evaluated via 1% agarose gel electrophoresis (Agilent Bioanalyser, 2100). For each sample, 1,000 ng of total RNA was utilized for cDNA synthesis employing the SuperScript first-strand synthesis system in a 20 µL reaction volume to standardize gene expression levels. The resulting cDNA was stored at −20°C for subsequent analysis. The expression levels of immune-related genes were quantified by qPCR utilizing particular primers detailed in **Table 1**. qPCR was performed with Hieff^®^ qPCR SYBR Green Master Mix (YEASEN) according to the manufacturer’s instructions. β-actin was used as an internal reference gene. Relative mRNA abundances were calculated using the 2^−ΔΔCt^ method and normalized to β-actin. To quantify LMBRaV abundance, DNA was isolated from HK and gill tissues, and specific primers listed in **Table 1** were used. Viral loads were quantified by establishing a standard curve using a LMBRaV plasmid. The average values from gene duplicates in the samples were extrapolated via the standard curve to determine LMBRaV copy numbers.

**Table 1:** Gene-specific primers used for qPCR in this study.

Gene	Forward primer (5’-3’)	Reverse primer (5’-3’)	Accession number
*IgM*	CTCAATGACCCCCCCTAA	CAAGCCAAGACACCAAAA	MN871984.1
*IgT*	GAAGGTCAACAACGCTGAGTG	TGTTGCTGGTCACATCTAGTCC	MZ981731.1
*CD4*	GACTGGAGTGGCGGAAAGTGGAGG	TTTCATCTTCTACAAACGCAGACAACGG	XM_038711093.1
*CD8*	GGAAGGGGATCCTGTTGACA	CCAGCACTCGAAACCAGATG	XM_038696403.1
LMBRaV*-MCP*	TCTGTTACGGGTTCTGGCATC	CCAGCCAAGAGTTGAGCACAT	FR682503.1
*β-actin*	CCACCACAGCCGAGAGGGAA	TCATGGTGGATGGGGCCAGG	MH018565.1

### 2.5 Development of monoclonal antibody against LMBRaV MCP

The anti-LMBRaV mouse monoclonal antibody was produced by ABclonal. Female BALB/c mice were immunized with the LMBRaV MCP protein in Freund’s complete adjuvant (Sigma-Aldrich). Splenocytes from the immunized mice and SP2/0 myeloma cells were fused, positive hybrids were screened and subcloned 4 times. Monoclonal antibody was produced either from the supernatants of the hybridoma culture or from the ascites of BALB/c mice.

### 2.6 Histology, light microscopy and immunofluorescence microscopy studies

To assess the morphological changes in HK and gill tissues following viral infection, we employed a modified methodology. Histological slices of the dissected gills and HK were fixed in 4% neutral buffered paraformaldehyde for a minimum of 24 hours. The fixed tissues were subsequently dehydrated using a graded ethanol series, cleaned with xylene, and embedded in paraffin for histological examination. Sections of 5–7 μm in thickness were produced using a rotary microtome (HM 325 Manual Microtome, Germany) and subsequently stained with standard hematoxylin and eosin (H&E) for regular histological analysis. The stained sections were analyzed microscopically using an Olympus BX53 microscope and Axiovision software. To detect LMBRaV or IgM^+^ B cells, slices were incubated with monoclonal mouse anti-LMBRaV-MCP or mouse anti-largemouth bass IgM (Biogoethe Biotechnology (Wuhan) Co., Ltd, 1 μg/mL) for 2 hours at 37°C. Following three washes, slices were incubated with Alexa Fluor 488-conjugated AffiniPure Goat anti-mouse IgG at a concentration of 2.5 μg/mL for 40 minutes at room temperature to identify cells infected with LMBRaV or IgM^+^ B cells.

### 2.7 Flow cytometry

The leucocytes from largemouth bass gill and HK were obtained as we previously reported (39). Briefly, fish were anaesthetized with MS-222 and blood was collected from the caudal vein. To remove the blood in the gills, PBS-heparin was perfused through the heart until the gills were completely blanched. Then gill and HK tissues were obtained and mechanically disaggregated on a 100-μm cell shredder and the cell fraction was collected. The cell fractions from the above tissue treatments were pooled, washed with Dulbecco’s modified eagle medium (DMEM) and passed through a 100-μm nylon mesh. The resulting cell suspension was washed three times in fresh DMEM and layered over a 51/34% discontinuous Percoll gradient. After 35 min of centrifugation at 400 g, leucocytes lying at the interface of the gradient were collected and washed with DMEM medium. In flow cytometry analysis of B cells in the gill and HK tissues, leukocyte suspensions were incubated with monoclonal mouse anti-largemouth bass IgM (1 µg/mL) on ice for 1 hour. Following three washes, Alexa Fluor 488-conjugated AffiniPure Goat anti-mouse IgG (1 µg/mL each) was administered and incubated for 45 minutes at 4°C to identify IgM^+^ B cells. Following three washes, stained leucocytes were examined using a CytoFLEX flow cytometer (Beckman Coulter) and evaluated with FlowJo software (Tree Star).

### 2.8 SDS-PAGE and Western blot

To obtain gill mucus, blood in the gills was first removed by perfusion with PBS–heparin through the heart until the gills were completely blanched. Gill arches were excised and rinsed with PBS three times to remove the remaining blood. Thereafter, gills were incubated for 12 h at 4 °C, with occasional shaking in protease inhibitor buffer (1 × PBS, containing 1 × protease inhibitor cocktail (Roche), 1 mM phenylmethylsulfonyl fluoride (Sigma); pH 7.2) at a ratio of 1 g of gill tissue per ml of buffer. The suspension (gill mucus) was collected into an Eppendorf tube, vigorously vortexed and centrifuged at 400 g for 10 min at 4 °C to remove trout cells. Serum and gill mucus samples were analyzed using 4–15% SDS-PAGE Ready Gel (Bio-Rad) under non-reducing conditions. The gels were transferred to PVDF membranes (Bio-Rad) for Western blot analysis. Subsequently, the membranes were blocked with 5% skim milk and treated with anti-largemouth bass IgM (mouse monoclonal antibody) followed by incubation with HRP-conjugated anti-mouse IgG (Invitrogen). Immunoreactivity was identified using an improved chemiluminescent reagent (Advansta) and analyzed with the GE Amersham Imager 600 Imaging System (GE Healthcare). The gel images obtained were examined using ImageQuant TL software (GE Healthcare). Subsequently, the IgM concentration was quantified by correlating the measured signal intensity values with a standard curve established for each blot with known quantities of pure largemouth bass IgM.

### 2.9 ELISA for detecting virus-specific IgM titers

The concentration of LMBRaV-specific IgM was assessed by indirect ELISA. To purify the LMBRaV, the aforementioned LMBRaV suspension was ultracentrifuged over a 20% sterile sucrose layer at 100,000× *g* for 2 h at 4 °C by using the Optima L-100XP (Beckman Coulter) ultracentrifuge. The resulting pellet (purified LMBRaV) was resuspended in PBS and its total protein concentration was determined by the Bradford protein assay according to the manufacturer’s instructions (Quick Start™ Bradford Protein Assay kit, Bio-Rad). High-binding 96-well microplates (Thermo Fisher Scientific) were incubated overnight at 4°C with 50 µL of pure viral antigen (10 µg/mL) diluted in phosphate-buffered saline (PBS) each well. The plates were subsequently incubated with 200 µL per well of 1% bovine serum albumin (BSA) in PBS for 1 hour at room temperature to inhibit non-specific binding. Subsequent to blocking, plates were rinsed thrice with PBST (phosphate-buffered saline supplemented with 0.05% Tween-20). Serum or gill mucus was introduced to the wells following gradient dilution in PBS, incubated at 4°C for 2 hours, and subsequently rinsed three times with PBST. 50 µL of mouse anti-largemouth bass IgM monoclonal antibody (1 μg/mL) diluted in phosphate-buffered saline containing 1% BSA was added to each well and incubated for 1 hour at room temperature. Subsequent to incubation, plates were subjected to five washes with PBST. Bound antibodies were identified through incubation with HRP Goat Anti-Mouse IgG (H + L) (0.5 μg/mL) in PBS at ambient temperature for 45 minutes. 100 µL of TMB substrate solution (Thermo Fisher Scientific) was dispensed into each well, and the reaction was permitted to run for 20 minutes in the dark at ambient temperature. 50 µL of 1 M sulfuric acid were added to each well to terminate the enzymatic reaction. Absorbance was quantified at 450 nm utilizing a microplate reader (Bio-Rad).

### 2.10 Statistical analysis

Data are presented as mean ± standard error of the mean (SEM). The examination of group differences employed the Student’s *t*-test utilizing Prism version 9.0 from GraphPad. Differences were deemed statistically significant at *P* < 0.05 or below.

## 3 Results

### 3.1 Construction and evaluation of a viral infection model in largemouth bass

**Figure 1:**
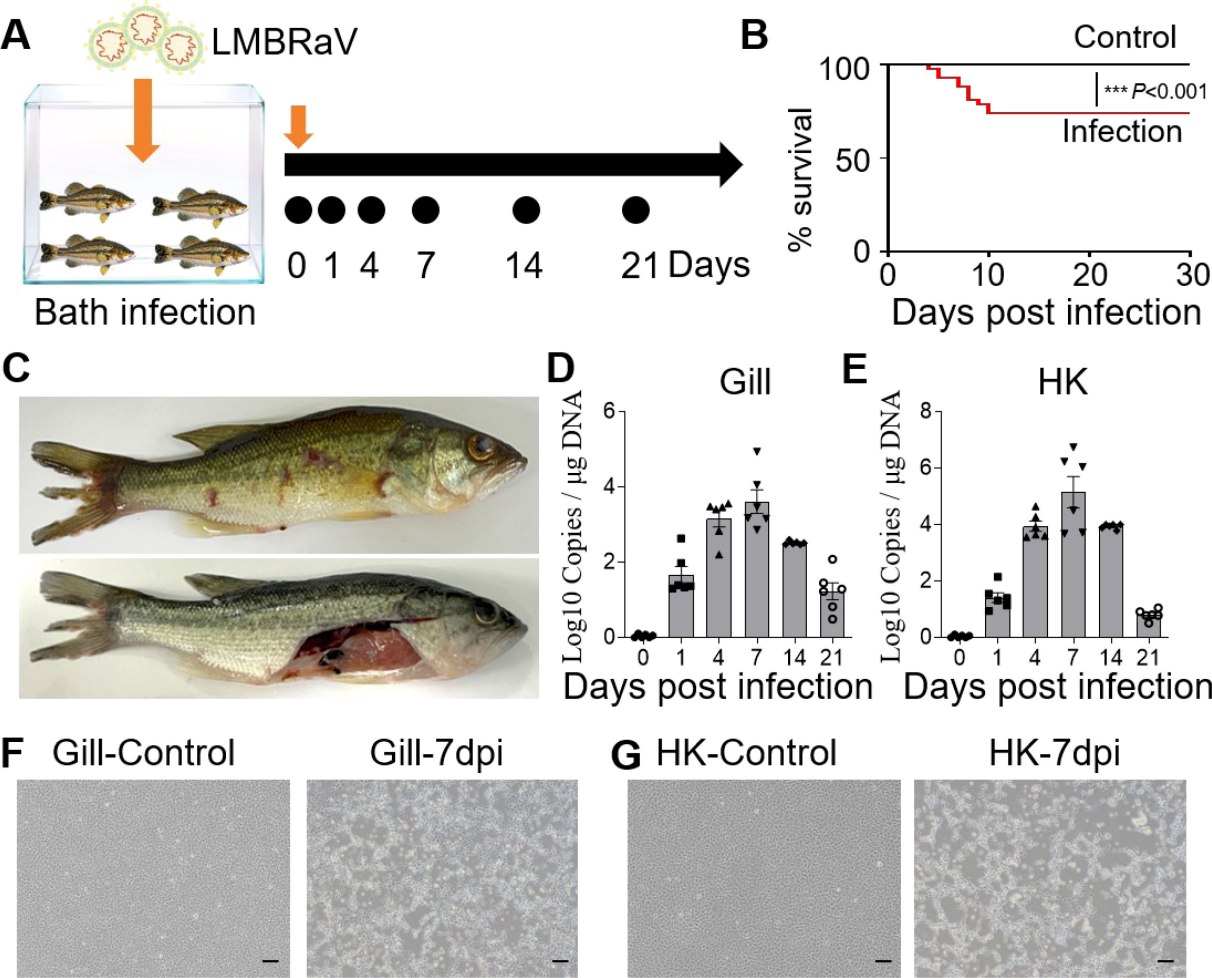
Construction of LMBRaV infection model in largemouth bass. **(A)** Diagram of LMBRaV infection strategy via bath immersion. **(B)** Cumulative survival rate of control and virus-infected fish. **(C)** Clinical signs of virus-infected fish at 7 dpi. **(D, E)** Fish tissues collected at 1, 4, 7, 14, and 21 dpi were analyzed for LMBRaV-MCP gene copies (Log10) using qPCR. The viral loads were detected in the fish gill **(D)**, and HK tissues **(E)** (*n* = 6). **(F, G)** The CPE of EPC cells after culturing with the supernatant of gill **(F)** and HK **(G)** homogenates from control and 7 dpi fish. Scale bars, 200 μm. ***p<0.001.

Largemouth bass were subjected to a 4-hour bath exposure to LMBRaV, and samples were collected at various time points (**Figure 1A**). Fish mortality was monitored daily. As shown in **Figure 1B**, mortality began on the 4th dpi and continued until the 10th day. By the end of this period, the cumulative mortality rate had reached 26%. At 7 dpi, infected fish exhibited symptoms such as fin rot, skin hemorrhaging, and a pale liver (**Figure 1C**). Viral loads in the gill and HK tissues were measured at 0, 1, 4, 7, 14, and 21 dpi using qPCR to detect the MCP gene of LMBRaV (**Figures 1D, E**). The highest viral loads in the gill and HK tissues were observed at 7 dpi, with elevated levels persisting until 14 dpi. Notably, the viral loads in the HK were higher than those in the gill at 4, 7, and 14 dpi. Furthermore, EPC cells incubated with homogenate supernatants from infected gill or HK tissues showed clear CPE compared to the controls (**Figures 1F, G**). These findings indicate that LMBRaV successfully invaded both the gill and HK tissues.

### 3.2 Histopathological changes and gene expression in infected fish

**Figure 2:**
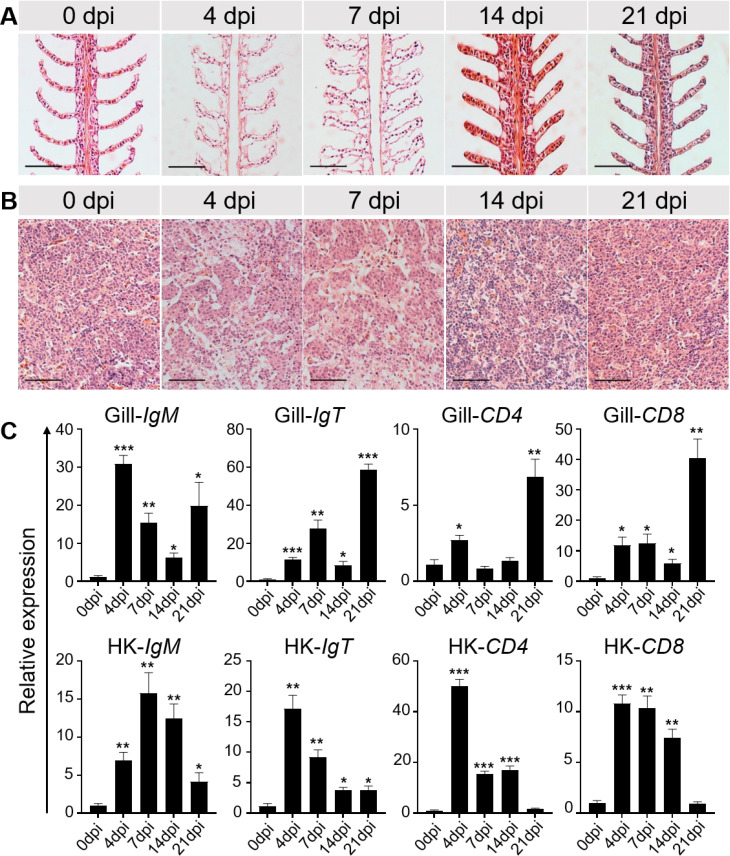
Histopathological changes and immune-related gene expressions in the gill and HK tissues of largemouth bass after LMBRaV infection. **(A, B)** H&E staining of the gill **(A)** and HK **(B)** from control and experimental fish infected with LMBRaV after 4, 7, 14, and 21 dpi. Scale bars, 50 μm. **(C)** Transcript levels of adaptive immune-related genes in gill and HK of control and LMBRaV−infected fish measured at 4, 7, 14, and 21 dpi (*n* = 6). Statistical differences were evaluated by unpaired Student’s *t*−test. *p<0.05, **p<0.01, ***p<0.001.

This study systematically analyzed the histopathological changes and gene expression profiles in the gill and HK tissues of LMBRaV-infected fish. H&E staining revealed significant pathological alterations caused by the viral infection. At 4 and 7 dpi, the lamellar epithelium of the gills showed signs of lifting, and edema was observed in the secondary gill lamellae (**Figure 2A**). The width to length ratio of secondary lamellae of gill tissue was significantly increased at 4, 7, and 14 dpi (**Supplementary Figure S1A**). Additionally, blood volume in the vascular axis of primary filaments was significantly reduced compared to the control group (**Figure 2A**). In the HK tissues, cellular degeneration led to enlarged cell gaps (**Figure 2B**, **Supplementary Figure S1B**). Notably, by 14 and 21 dpi, the tissues showed a gradual return to normal (**Figures 2A, B**). To further investigate the immune response, the expression levels of adaptive immune genes in the gill and HK tissues were measured at all sampling time points. In the gills, IgM gene expression was elevated at 4 and 21 dpi, while IgT, CD4, and CD8 gene expression peaked at 21 dpi. In the HK tissues, the highest expression levels of these adaptive immune genes were observed at 4 or 7 dpi (**Figure 2C**).

### 3.3 Reduced mortality and decreased tissue viral loads following secondary viral infection

**Figure 3:**
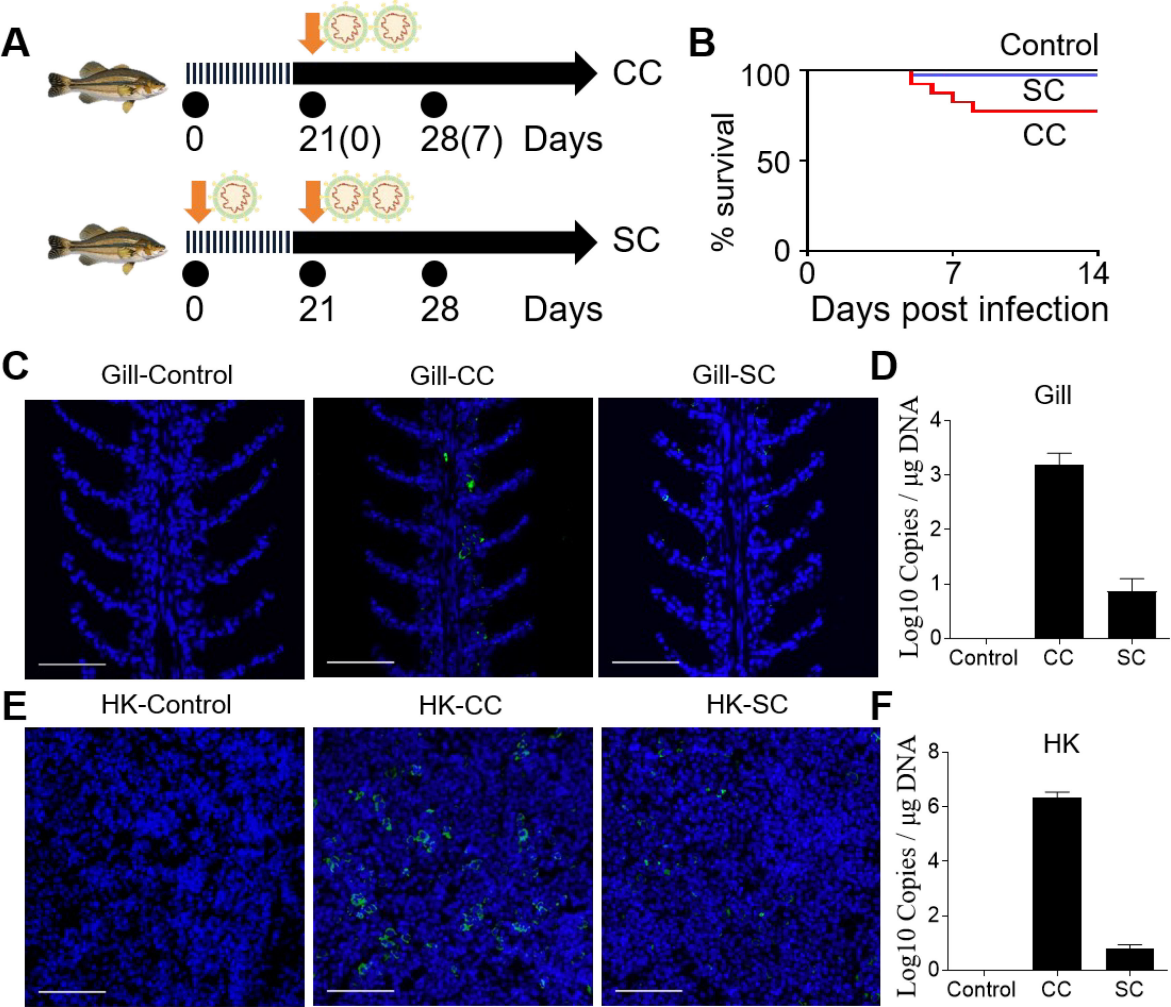
Survivor fish previously infected with LMBRaV become resistant to re-infection when challenged with a high dose of the virus. **(A)** Strategy to obtain the different groups of control and survivor fish. Control group was healthy fish without virus infection. At 21 days, fish were immersed in a solution containing 80 mL of LMBRaV (3.5 × 10^7^ TCID50) in 20 L of aerated water, and 7 days post-challenge fish (control challenge of CC group) were sacrificed for sampling. To generate 21 dpi survivor fish, fish were immersed in a solution containing 40 mL of LMBRaV (3.5 × 10^7^ TCID50) in 20 L of aerated water, and at 21 days after the first infection, fish were challenged with double concentration of LMBRaV, and 7 days post-challenge fish (21dpi-S challenge of SC group) were sacrificed for sampling. **(B)** Percentage survival of control, CC, and SC fish groups. **(C, E)** Viral particles were detected with the anti-LMBRaV-MCP protein mAb (green) in gill **(C)** and HK tissues **(E)** from control, CC, and SC fish. Scale bars, 50 μm. **(D, F)** The LMBRaV-MCP gene expression in gill and HK of control, CC, and SC fish groups were quantified using qPCR (*n* = 6).

To evaluate the adaptive immune response of largemouth bass to viral infection, fish that survived the initial infection were rechallenged at 21 dpi with a double dose of LMBRaV (21 dpi-S challenge, SC group). Simultaneously, a control group (control challenge, CC group) was infected with the same virus concentration (**Figure 3A**). The results showed that the mortality rate in the SC group was significantly lower than in the CC group (**Figure 3B**). Viral loads in gill and HK tissues were also quantified in the control, CC, and SC groups. Fluorescence microscopy revealed a markedly reduced viral signal in the gill and HK tissues of the SC group compared to the CC group. In the CC group, widespread viral antigen distribution indicated active infection, whereas the SC group exhibited minimal viral antigen presence, suggesting effective immune clearance (**Figures 3C, E**). qPCR analysis further confirmed that the viral DNA levels in the SC group were significantly lower than in the CC group. Specifically, viral DNA in the gill tissues of the SC group was reduced by 73%, whereas the HK tissues exhibited an 88% reduction (**Figures 3D, F**).

### 3.4 IgM^+^ B cells and IgM responses to LMBRaV

**Figure 4:**
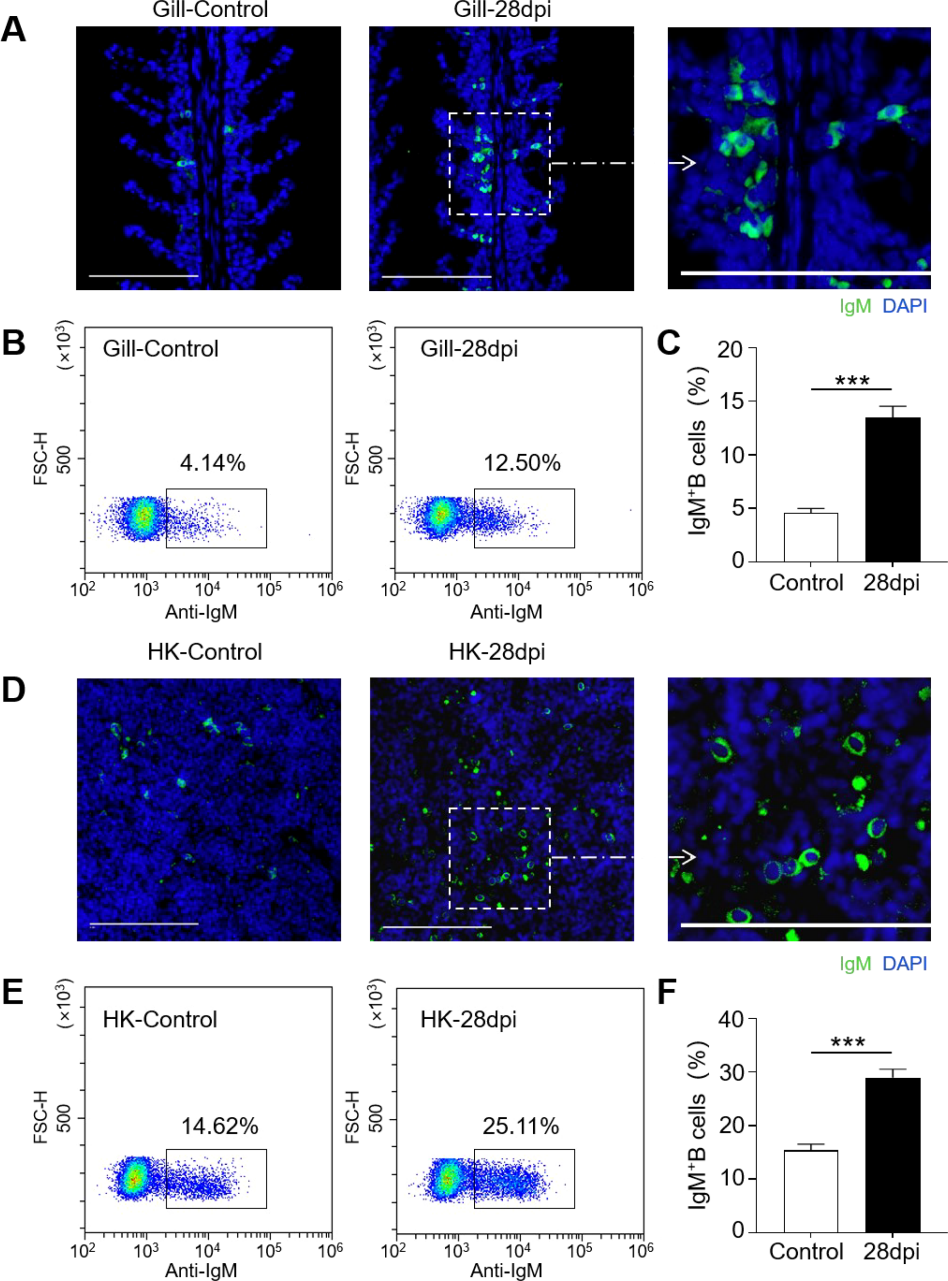
Increases of IgM^+^ B cells in the gill and HK tissues of largemouth bass infected with LMBRaV. **(A, D)** Differential interference contrast (DIC) images of immunofluorescence staining on largemouth bass gill **(A)** and HK **(D)**paraffin-sections from control and 28 dpi fish, stained for IgM (green); Nuclei were stained with DAPI (blue). Scale bars, 100 μm. **(B, E)** Representative flow cytometry dot-plots showing ratios of IgM^+^ B cells in gill **(B)** and HK **(E)** leukocytes of control and 28 dpi fish. **(C, F)** Percentage of IgM^+^ B cell populations in gill **(C)** and HK **(F)** of control and 28 dpi fish (*n* = 6). Statistical differences were evaluated by unpaired Student’s *t*−test. ***p<0.001.

**Figure 5:**
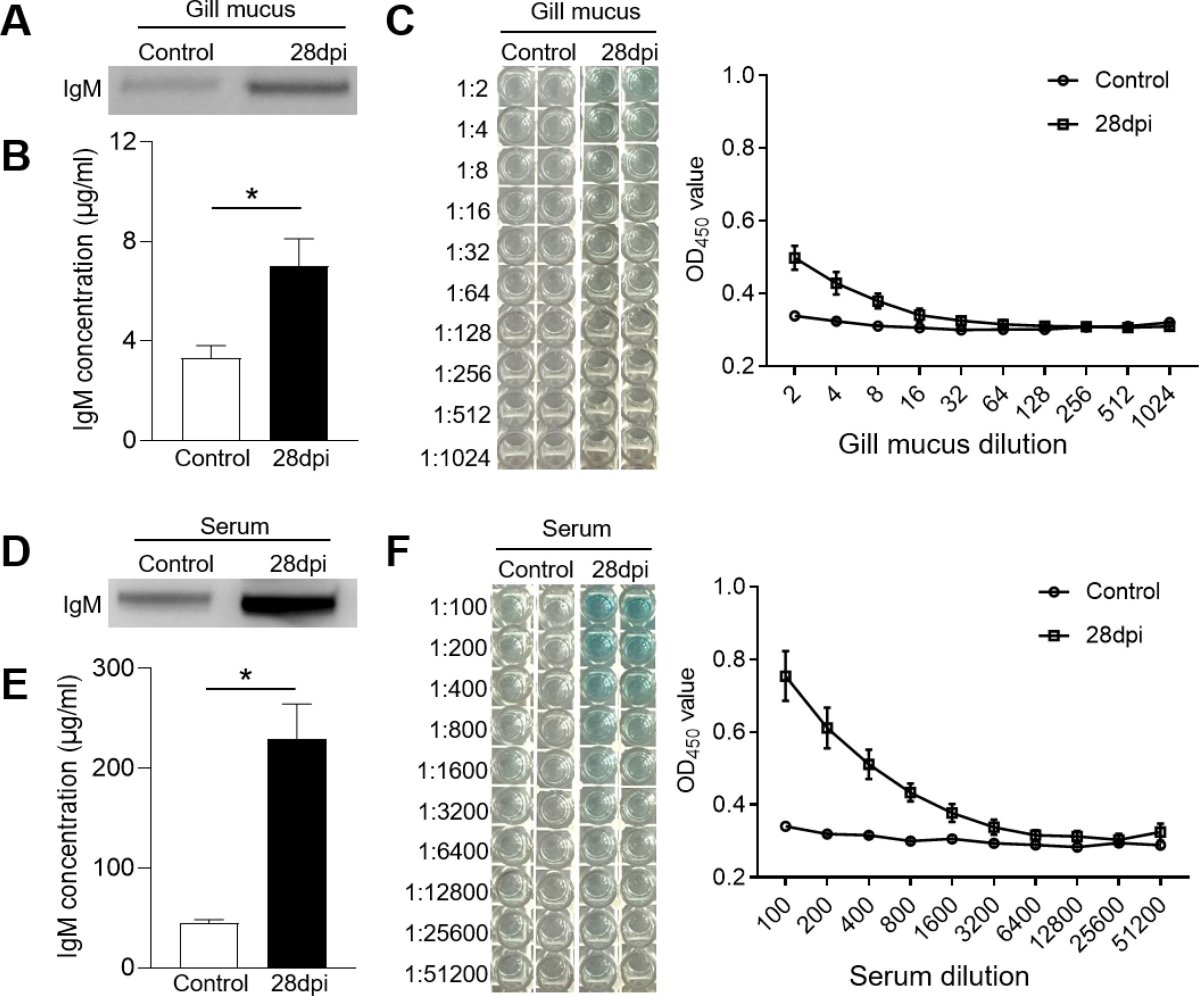
IgM responses in the gill mucus and serum from 28 dpi fish. **(A, D)** Western blot analysis of total IgM protein in gill mucus **(A)** and serum **(D)** of control and 28 dpi fish. **(B, E)** The concentration of IgM protein in gill mucus **(B)** and serum **(E)** of control and 28 dpi fish (*n* = 6). **(C, F)** ELISA analysis and the titers of specific IgM binding to LMBRaV in gill mucus **(C)** and serum **(F)** of control and 28 dpi fish. OD 28 dpi/OD control >2 is considered as significant potency. *P < 0.05.

The IgM^+^ B cells and IgM responses in the gill and HK tissues of largemouth bass were assessed following the secondary viral infection. Immunofluorescence staining indicated a sparse presence of IgM^+^ B cells in the gills of control fish (**Figure 4A**). However, by 28 dpi, the number of IgM^+^ B cells had increased significantly, from 4.14% to 12.50% (**Figures 4B, C**). Notably, the HK tissue contained a higher number of IgM^+^ B compared to the gills (**Figure 4D**). Following viral infection, IgM^+^ B cell proliferation in the HK increased substantially from 14.62% to 25.11% (**Figures 4E, F**). Additionally, IgM protein concentration in the gill mucus of the 28 dpi group was approximately double that of the control fish (**Figures 5A, B**). In the serum, IgM protein levels increased approximately five-fold in 28 dpi fish (**Figures 5D, E**). The significant increases in IgM^+^ B cells and IgM protein levels in the gill and HK tissues of surviving fish suggest the generation of virus-specific IgM immune responses. To confirm this, virus-specific IgM titers in the gill mucus and serum were measured using ELISA. The IgM in the serum and mucus displayed high reactivity toward LMBRaV antigens, as indicated by optical density (OD) values two-fold higher than the control OD. As shown in **Figure 5C**, gill mucus from 28 dpi fish exhibited higher OD values than the control when diluted to 1/2 and 1/4, though the difference was not statistically significant. In the serum, LMBRaV-specific IgM was detected at a dilution of 1:200 in the 28 dpi group (**Figure 5F**).

### 3.5 LMBRaV-specific IgM has a viral neutralizing capacity

**Figure 6:**
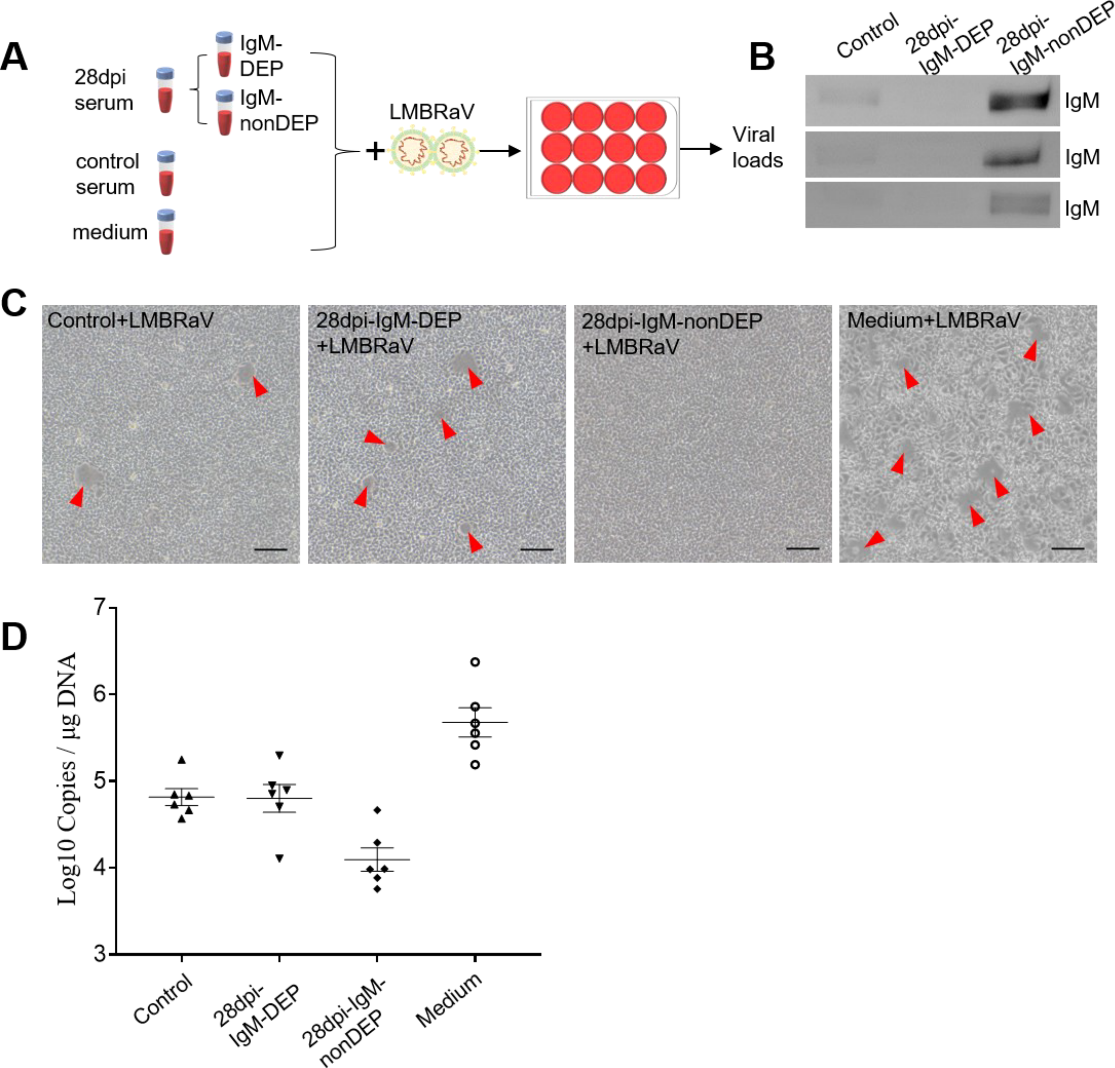
Viral neutralization exerted by LMBRaV-specific IgM from serum of 28 dpi fish. **(A)** Scheme of the experimental strategy. Magnetic protein G protein was incubated with anti-largemouth bass IgM mAbs to generate protein G beads coated with anti-IgM mAbs. IgM from 28 dpi serum was depleted by incubating the serum with protein G beads coated with anti-IgM mAbs. Thereafter, LMBRaV was pre-incubated with medium alone or serum derived from control and 28 dpi fish, and each of these LMBRaV-containing serum were added to EPC cells. Three days after addition of the different serum or medium alone to EPC cells, these four groups of EPC cell treatments (including: Medium + LMBRaV, Control + LMBRaV, 28 dpi-IgM-nonDEP + LMBRaV, and 28 dpi-IgM-DEP + LMBRaV) were analyzed for CPE of LMBRaV on EPC cells and viral loads. **(B)** Western blot analysis of IgM protein in serum of control, 28 dpi fish (IgM-DEP and IgM-nonDEP). **(C)** CPE of EPC cells in the four groups. **(D)** The viral loads of LMBRaV in the four groups of EPC cells (*n* = 6).

Our findings demonstrate that LMBRaV infection leads to a significant increase in LMBRaV-specific IgM titers in the serum (**Figure 5**), and fish re-exposed to LMBRaV exhibited enhanced resistance to the virus (**Figure 3**). Therefore, we hypothesized that LMBRaV-specific IgM may neutralize the virus in the serum. To test this, LMBRaV was incubated for 1 hour with different solutions, including a medium control, serum from 28 dpi fish, and serum from uninfected controls. To assess the role of IgM, we first depleted IgM from the serum of 28 dpi fish, creating IgM-depleted serum (28 dpi-IgM-DEP serum) (**Figures 6A, B**). After incubation, the four LMBRaV-containing sera were tested on EPC cells, and both CPE and LMBRaV loads were assessed in the EPC cultures. The viability of EPC cells treated with serum from 28 dpi fish was higher than that of cells treated with serum from control fish (**Figure 6C**), indicating a protective effect of the serum. However, when EPC cells were treated with 28 dpi-IgM-DEP serum, their viability was lower than that of cells treated with uninfected fish serum, suggesting that the protective effect is mediated by LMBRaV-specific IgM (**Figure 6C**). This was further corroborated by the significant reduction in the expression of the LMBRaV-MCP gene in EPC cells treated with serum from 28 dpi fish, which returned to control levels when IgM was depleted (in 28 dpi-IgM-DEP serum) (**Figure 6D**). Collectively, these results strongly suggest that serum IgM plays a crucial role in neutralizing LMBRaV.

## 4 Discussion

B cells and Igs are key components of the adaptive immune system in vertebrates, acting as effector molecules that mediate immune responses. In teleost fish, IgM is the predominant immunoglobulin isotype, playing a central role in both systemic and mucosal immunity. Previous research has underscored the importance of IgM in the humoral immune response to various pathogens, including viruses. However, the specific antiviral function of IgM in different tissues, particularly in the gills and HK of largemouth bass, remains largely underexplored. Therefore, this study aimed to investigate the role of IgM in the immune responses of the gills and HK during a ranavirus infection.

To address this, we simulated a natural infection environment by exposing largemouth bass to LMBRaV via immersion. Similar to previous studies, the infected fish exhibited clinical symptoms such as fin rot, skin hemorrhaging, and pale liver (33). However, compared to fish infected via intraperitoneal injection in other studies, our results showed a delayed onset of mortality and symptoms in the immersion-infected fish (40, 41). This suggests that mucosal barriers may play a critical role in preventing viral invasion. Additionally, we monitored viral loads in the gill and HK tissues at various time points post-infection. Notably, viral loads peaked at 7 dpi in both tissues, with higher concentrations in the HK than in the gills, suggesting a greater susceptibility of the HK to viral infection. Similar patterns have been observed in other virus-infected fish models (42, 43).

Importantly, severe pathological changes were observed in the gill and HK tissues at 4 and 7 dpi. The secondary gill lamellae were swollen, and the HK tissues showed enlarged cell gaps, indicative of a strong immune response. The swelling of the gill lamellae suggests an inflammatory reaction, likely due to immune cell infiltration and increased vascular permeability. These observations align with findings from Zawisza et al. (44), who reported pronounced gill inflammation and edema in virus-infected fish. The enlarged cell gaps in the HK may reflect tissue damage caused by viral replication, a phenomenon also noted by Sun et al. (45) in teleosts. These changes not only highlight the direct impact of viral infection but also suggest the activation of immune mechanisms aimed at controlling the virus.

We further examined the expression of several immune-related genes in both gill and HK tissues. In the gills, the expression of IgM, IgT, CD4, and CD8 was significantly elevated at 4 and 21 dpi, with a peak at 21 dpi for all genes except IgM. Similar findings have been reported in other virus-infected fish models (46, 47), implying that the adaptive immune response is crucial for gill resistance during the later stages of infection. In contrast, in the HK tissues, the expression of these adaptive immune genes peaked at 4 or 7 dpi, suggesting a rapid early immune response essential for controlling initial viral replication. This pattern suggests that while the gills sustain an adaptive immune response, the HK primarily functions in acute immune activation, swiftly mobilizing immune cells and factors to combat the virus in the early stages of infection. In mammals, CD4 and CD8 were marker genes of CD4^+^ and CD8^+^ T cells, which have been demonstrated to be crucial in infectious immunity, including efficient clearance of pathogens, helping B cell response and antibody production, rapid control of reinfection, and providing long-term protection by memory formation (48). Similarly, it has been reported that CD4 and CD8 mRNA significantly up-regulated during tuberculin injection and the infection of *Edwardsiella tarda* and viral hemorrhagic septicemia virus in flounder (*Paralichthys olivaceus*) (49, 50). Additionally, fish CD4^+^ T cells were shown to be involved in regulating the immune response of B lymphocytes (51). In summary, our results suggest that adaptive immune responses may play an important function in the resistance of largemouth bass to viral infections.

Secondary stimulation in fish that survived the initial infection involved administering higher doses of the virus, resulting in a significant reduction in mortality and viral loads in the HK and gill tissues. This observation, along with our earlier findings on immune gene expression, underscores the importance of the adaptive immune response in these tissues during the later stages of viral infection.

Using an anti-largemouth bass IgM monoclonal antibody, we assessed the number of IgM^+^ B cells in the gill and HK tissues of both control and 28 dpi fish. In line with previous studies in rainbow trout, where IgM^+^ B cells increased significantly in HK tissues infected with parasites (40), bacteria (52), and viruses (15), we observed a notable rise in IgM^+^ B cells from 14.62% in control fish to 25.11% at 28 dpi in largemouth bass. Interestingly, the IgM^+^ B cells were also significantly enriched in the gills following viral infection, differing from reports in fish gills infected with parasites (26) or bacteria (47), where an accumulation of IgT^+^, but not IgM^+^ B cells, was detected. This suggests that IgM^+^ B cells play a crucial role in resisting viral invasion, not only in systemic immune tissues but also in mucosal immune tissues. Similar findings were reported by Yu et al. (29) in carp, where viral infection of the intestine led to a marked increase in IgM^+^ B cells. Furthermore, it has been documented that two distinct subsets of IgM^+^ cells, namely IgM^+^ lymphocytes and IgM^+^ myeloid cells, have been identified in grass carp (*Ctenopharyngodon idella*) and tilapia (*Oreochromis niloticus*) (53, 54). Additionally, IgM^+^ myeloid cells have been observed to exhibit morphological characteristics akin to those of plasma cells, which could potentially be induced by CD40L (54, 55). To gain a deeper insight into the function of IgM^+^ B cells in largemouth bass, further studies are required to focus on the distinct roles of IgM^+^ lymphocytes and IgM^+^ myeloid cells.

Immunoglobulins, produced and secreted by B cells, directly interact with viruses during the antiviral immune response (56). Our experimental results showed a substantial elevation of IgM levels in both gill mucus and serum after viral infection, which coincided with a significant increase in B cells. Virus-specific IgM antibodies were also detected in both gill mucus and serum, although serum titers were significantly higher. This suggests that IgM primarily functions in systemic immune tissues, a conclusion consistent with the findings of Zhang et al. (10). While our earlier results showed a reduced viral load in the gills following secondary infection, the virus-specific IgM titers in gill mucus were not significantly elevated, indicating that other immunoglobulin types or cellular immune mechanisms may play a more prominent role. Further research is needed to validate this hypothesis.

Although IgM responses in teleost plasma have been widely studied, characterized by high titers in response to infection or vaccination, few studies have specifically explored the functional role of IgM in pathogen interactions. Although IgT has been shown to neutralize viruses in teleosts (15), the role of IgM in viral neutralization has remained unclear. To address this, we conducted a depletion experiment where IgM was removed from serum. The results revealed that IgM-depleted serum, when incubated with EPC cell cultures, exhibited a significant reduction in its ability to protect EPC cells from viral infection. These findings support the hypothesis that fish IgM plays a previously unrecognized role in viral neutralization. Similar IgM-mediated viral neutralization has been demonstrated in humans, such as ultrapotent ZIKV-specific IgM neutralization (57), and a similar blocking mechanism has been observed in mice (58). Further research is needed to uncover the exact mechanisms through which IgM neutralizes viruses in fish, which will deepen our understanding of IgM’s role in the immune response to viral infections.

In conclusion, our study highlights the significant roles of IgM^+^ B cells and IgM in combating LMBRaV infection in both systemic and mucosal immune tissues. LMBRaV infection induces considerable pathological changes and triggers robust immune responses. Secondary viral infection led to an accumulation of IgM^+^ B cells and an increase in IgM protein concentration, along with high titers of virus-specific IgM. This immune response likely contributes to the reduced viral load and enhanced survival rates observed in infected fish. Moreover, similar to IgT, we found that fish IgM has the capacity to neutralize viruses. These results underscore the critical role of IgM in antiviral immunity, suggesting its potential as a target for improving disease resistance in aquaculture.

## Data Availability

The raw data supporting the conclusions of this article will be made available by the authors, without undue reservation.

## Supplementary material

Supplementary Figure S1 The statistical analysis of histopathological changes in gill and HK tissues. **(A)** The width to length ratio in secondary lamellae of gill tissue. **(B)** The area of the cellular vacuole of HK tissue. **p*<0.05, ****p*<0.001.
