# Supplementary material for: Immunoglobulin M response in largemouth bass (Micropterus salmoides) following ranavirus infection
Source: Front Immunol. 2025 Jan 29;16:1515684. doi: 10.3389/fimmu.2025.1515684 (PMC11814181; doi:10.3389/fimmu.2025.1515684)
Supplement: Supplementary Figure S1 — The statistical analysis of histopathological changes in gill and HK tissues. (A) The width to length ratio in secondary lamellae of gill tissue. (B) The area of the cellular vacuole of HK tissue. *p<0.05, ***p<0.001. [file DataSheet1.docx]

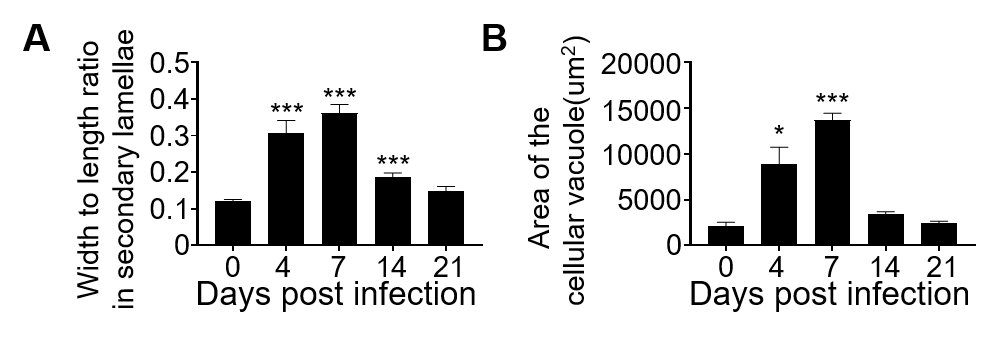


**Figure S1.** The statistical analysis of histopathological changes in gill and HK tissues. (A) The width to length ratio in secondary lamellae of gill tissue. (B) The area of the cellular vacuole of HK tissue. **p*<0.05, ****p*<0.001.
